# Low-intensity pulsed ultrasound improves behavioral and histological outcomes after experimental traumatic brain injury

**DOI:** 10.1038/s41598-017-15916-2

**Published:** 2017-11-14

**Authors:** Wei-Shen Su, Chun-Hu Wu, Szu-Fu Chen, Feng-Yi Yang

**Affiliations:** 1 Department of Biomedical Imaging and Radiological Sciences, National Yang-Ming University, Taipei, Taiwan; 2 Graduate Institute of Life Sciences, National Defense Medical Center, Taipei, Taiwan; 3 Departments of Physiology and Biophysics, National Defense Medical Center, Taipei, Taiwan; 4 Department of Physical Medicine and Rehabilitation, Cheng Hsin General Hospital, Taipei, Taiwan; 5 Biophotonics and Molecular Imaging Research Center, National Yang-Ming University, Taipei, Taiwan

## Abstract

The purpose of this study was to investigate the neuroprotective effects of low-intensity pulsed ultrasound (LIPUS) on behavioral and histological outcomes in a mouse model of traumatic brain injury (TBI). Mice subjected to controlled cortical impact injury were treated with LIPUS in the injured region daily for a period of 4 weeks. The effects of LIPUS on edema were observed by MR imaging in the mouse brain at 1 and 4 days following TBI. Brain water content, blood-brain barrier permeability, histology analysis, and behavioral studies were performed to assess the effects of LIPUS. Two-way analysis of variance and Student t test were used for statistical analyses, with a significant level of 0.05. Treatment with LIPUS significantly attenuated brain edema, blood-brain barrier permeability, and neuronal degeneration beginning at day 1. Compared with the TBI group, LIPUS also significantly improved functional recovery and reduced contusion volumes up to post-injury day 28. Post-injury LIPUS treatment reduced brain edema and improved behavioral and histological outcomes following TBI. The neuroprotective effects of LIPUS may be a promising new technique for treating TBI.

## Introduction

Traumatic brain injury (TBI) is characterized by damage to the brain as a result of a mechanical force, a rapid acceleration-deceleration movement, or a blast wave. TBI triggers a complex cascade of inflammatory responses that cause tissue injury and behavioral impairment^1^. The initial inflammatory response after TBI results in blood-brain barrier disruption (BBBD) and neuronal damage^2,3^. BBBD is considered to be the major cause of vasogenic brain edema and subsequent brain injury^4,5^. It has become increasingly evident that the development of cerebral edema with brain swelling leads to high mortality and morbidity in TBI patients^6,7^. Thus, attenuating the permeability of the BBB has been identified as a promising method for controlling cerebral edema and associated brain swelling.

Neuroprotection is a potential approach for the treatment of TBI, but no neuroprotective agents have been shown to be effective for such injuries in clinical trials^8^. One important barrier is that many drugs cannot be sufficiently delivered to the injured brain due to the BBB. Focused ultrasound with microbubbles can locally disrupt the BBB for enhanced drug delivery, but this technology has almost always been found to be associated with sterile inflammatory response and damage can occur under inertial cavitation conditions^9–11^. It has been demonstrated that low-intensity pulsed ultrasound (LIPUS) accelerates bone healing and axonal regeneration after injury^12,13^. Ultrasound holds promise as a powerful neurostimulation tool^14,15^. In particular, LIPUS alone may be able to stimulate neuronal activity and enhance the levels of neurotrophic factors^16,17^. Furthermore, one study suggests that the application of LIPUS in the early stages of TBI will effectively enhance the recovery of the BBB and alleviate the brain edema^18^. However, the neuroprotective effects of LIPUS exposure on TBI have not been established.

Consequently, in the present study, we investigated the hypothesis that LIPUS stimulation may ameliorate brain edema, functional impairment, and neuronal damage after experimental TBI in mice. Our results have revealed novel neuroprotective effects of LIPUS on TBI, indicating the possibility that LIPUS may be useful in the treatment of brain injuries.

## Materials and Methods

### Animals and Surgical Procedures

All animal experiments were performed according to the guidelines of and were approved by the Animal Care and Use Committee of National Yang-Ming University. The animals were blindly randomized to different treatment groups by using computer-generated random numbers. All outcome measurements described below were also performed in a blinded manner. The TBI model was induced by controlled cortical impact (CCI) injury in mice. Male C57BL/6 J mice (8 weeks old, about 22–25 g in weight) were intraperitoneally anesthetized with sodium pentobarbital (65 mg/kg; Rhone Merieux, Harlow, UK) and placed in a stereotaxic frame. A 5 mm craniotomy was performed over the right parietal cortex, centered on the coronal suture and 0.1 mm lateral to the sagittal suture, and injury to the dura was avoided. A CCI device (eCCI Model 6.3; Custom Design, Richmond, VA, USA) was used to perform unilateral brain injury by a pneumatic piston device with a rounded metal tip (2.5 mm in diameter) that was angled at 22.5° to the vertical so that the tip was perpendicular with the brain surface at the center of the craniotomy. A velocity of 4 m/s and a deformation depth of 2 mm below the dura were applied. The bone flap was immediately replaced and sealed, and the scalp was sutured closed. Mice were placed in a heated cage to maintain body temperature while recovering from anesthesia. Sham-operated mice received craniotomy as described before, but without CCI; the impact tip was placed lightly on the dura before sealing the wound. After the trauma or sham surgery, animals were housed under the conditions mentioned above.

### Pulsed Ultrasound Apparatus

**Figure 1:**
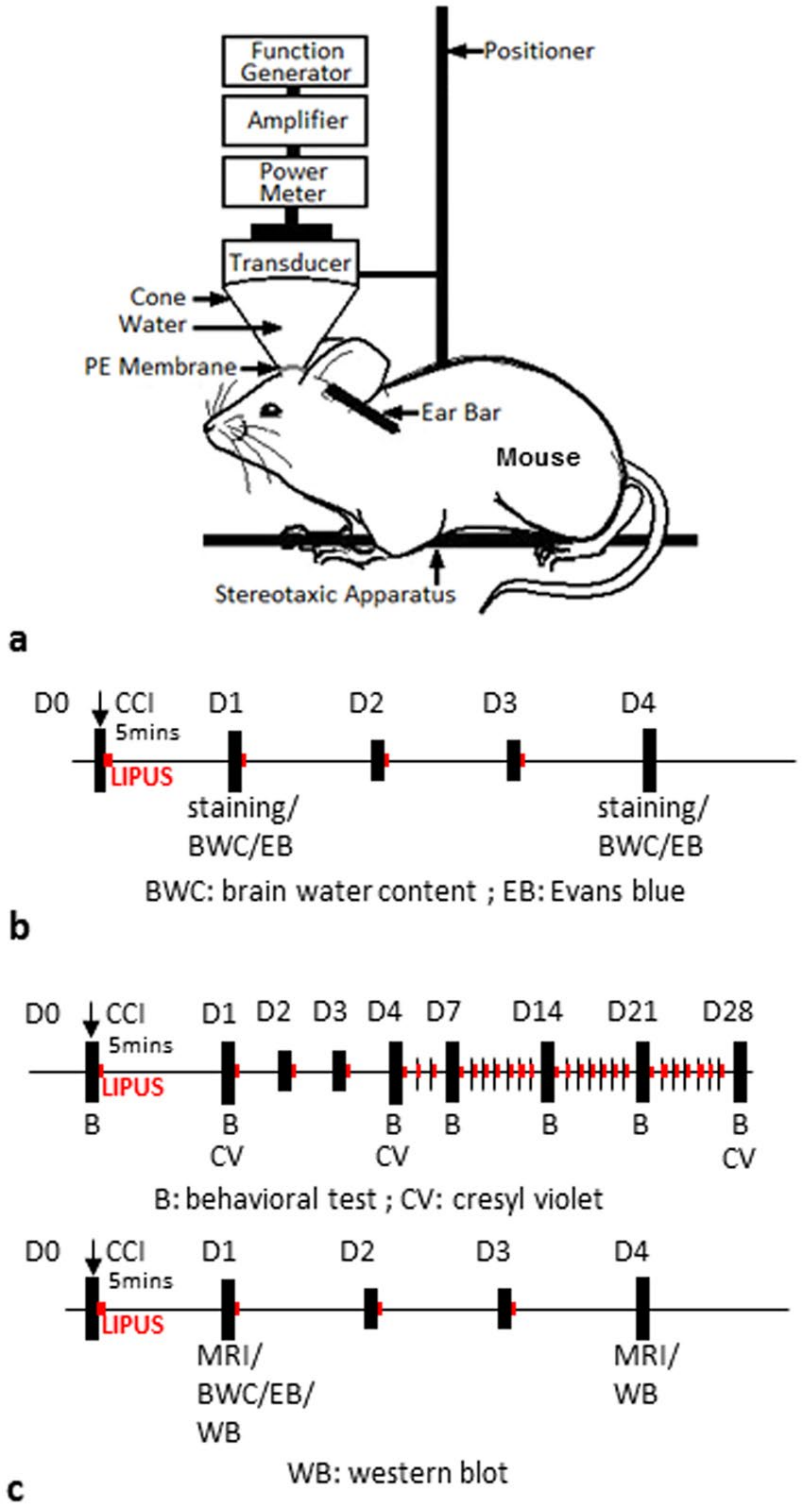
Experimental design. (**a**) Schematic diagram of low-intensity pulsed ultrasound setup. (**b**) LIPUS was performed daily from day 0 to day 3 (red point) in normal brain. (**c**) LIPUS was performed daily from day 0 to day 27 or day 3 (red point) in a TBI mouse.

The pulsed ultrasound setup was similar to that used in our previous study (Fig. 1a)^19^. LIPUS exposures were generated by a 1.0-MHz, single-element focused transducer (A392S, Panametrics, Waltham, MA, USA) with a diameter of 38 mm and a radius of curvature of 63.5 mm. The half-maximum of the pressure amplitude of the focal zone had a diameter and length of 3 mm and 26 mm, respectively. The transducer was applied with a duty cycle of 5% and a repetition frequency of 1 Hz. The transducer was mounted on a removable cone filled with deionized and degassed water whose tip was capped by a polyurethane membrane, and the center of the focal zone was about 2.0 mm away from the cone tip. The mice were anesthetized with isoflurane mixed with oxygen during the sonication procedure. The sonication was precisely targeted using a stereotaxic apparatus (Stoelting, Wood Dale, IL, USA). The acoustic wave was delivered to the targeted region in the injured cortical areas. A function generator (33220A, Agilent Inc., Palo Alto, USA) was connected to a power amplifier (500–009, Advanced Surgical Systems, Tucson, AZ) to create the US excitation signal. A power meter/sensor module (Bird 4421, Ohio, USA) was used to measure the input electrical power. LIPUS was applied for a sonication time of 5 min at an acoustic power of 0.51 W (corresponding to a spatial-peak temporal-average intensity (I_SPTA_) of 528 mW/cm^2^) 5 mins after TBI and subsequently daily for a period of 3 or 27 days (Fig. 1b,c). Mice were sacrificed for analysis at 1, 4, or 28 days. The intensity of the LIPUS exposures was selected based on data from our previous studies^17,20^ and a pilot study in which a sonication time of 5 min or 15 min at an acoustic power of 0.11 W or 0.51 W was tested; a sonication time of 5 min at an acoustic power of 0.51 W attenuated brain water content and there was no significant difference between the other two LIPUS-treated TBI groups and the non-treated TBI group (Table 1).

**Table 1:** Effects of different LIPUS treatment protocols on brain water content in TBI mice at 1 day.

	Sham	TBI	TBI + 0.11 W/5 min	TBI + 0.51 W/5 min	TBI + 0.51 W/15 min
BWC(%)	79.49 ± 0.18	84.58 ± 0.43^a^	85.37 ± 0.21^a^	82.25 ± 0.50^ab^	83.93 ± 1.03^a^

BWC: brain water content. a and b denote significantly different from sham and non-treated TBI group, respectively (^a,b^ *p* < 0.05; *n* = 4).

### Neurological Function Evaluation

Behavioral assessments (neurological severity scores (mNSS), rotarod, and beam walk) were performed before and at days 1, 4, 7, 14, 21, and 28 after CCI^21^. The mNSS includes a composite of motor, sensory, reflex, and balance tests. The mNSS rates neurological functioning on a scale of 0–18 from normal to maximal deficit (Table 2). In addition, mice were pretrained for 3 days for both the rotarod and beam walk tests. Moreover, three trials were recorded 1 h before CCI to determine baseline values. The rotarod task measures balance and motor activity. The speed of rotation was gradually accelerated from 6 to 42 rpm within 7 min. Each mouse was placed on a 3 cm rotating rod, and the latency to fall was recorded for all trials. The beam walk is used to evaluate fine motor coordination and function by measuring the ability of an animal to traverse an elevated beam^22^. The time for the mouse to traverse the beam (not to exceed 60 s) and the hindlimb performance as it crossed the beam (based on a 1 to 7 rating scale) were recorded. A score of 7 was given when animals traversed the beam with two or less footslips; 6 was given when animals traversed the beam with less than 50% footslips; 5 was given for more than 50% but less than 100% footslips; 4 was given for 100% footslips; 3 was given for traversal with the affected limb extended and not reaching the surface of the beam; 2 was given when animals were able to balance on the beam but not traverse it; 1 was given when animals could not balance on the beam^21^.

**Table 2:** Modified neurological severity score (mNSS).

**Motor tests**	
*Raising mice by tail (normal* = *0, maximum* = *3)*	
Flexion of forelimb	1
Flexion of hindlimb	1
Head moved >100 to vertical axis within 30 s	1
*Placing mice on floor (normal* = *0, maximum* = *3)*	
Normal walk	0
Inability to walk straight	1
Circling toward the paretic side	2
Falls down to paretic side	3
*Sensory tests (normal* = *0, maximum* = *2)*	
Placing test (visual and tactile test)	1
Proprioceptive test (deep sensation, pushing paw against table edge stimulate limb muscles)	1
*Beam balance tests (normal* = *0, maximum* = *6)*	
Balances with steady posture	0
Grasps side of beam	1
Hugs beam and 1 limb falls down from beam	2
Hugs beam and 2 limb falls down from beam, or spins on beam (>60 s)	3
Attempts to balance on beam but falls off (>40 s)	4
Attempts to balance on beam but falls off (>20 s)	5
Falls off, no attempts to balance on beam (<20 s)	6
*Reflex absence and abnormal movements (normal* = *0, maximum* = *4)*	
Pinna reflex (head shaken when auditory meatus is touched)	1
Corneal reflex (eye blink when cornea is lightly touched with cotton)	1
Startle reflex (motor response to a brief noise from clapping hands)	1
Seizures, myoclonus, myodystony	1
*Maximum points*	18
One point is given for an absent tested or for the animal’s inability to perform a task	
1–6 mild injury, 7–12 moderate, 13–18 severe injury	

### Brain Water Content Determination

Mice were sacrificed at day 1 and day 4, two time points associated with the maximum appearance of edema after TBI^23–25^. Brain water content was measured in a 4 mm coronal tissue section of the ipsilateral hemisphere 2 mm from the frontal pole. Brain samples were weighed on an electric analytical balance to obtain the wet weight and then dried at 100 °C for 24 h to obtain the dry weight. Brain edema was evaluated by measuring brain water content using the formula of (wet weight-dry weight)/wet weight × 100%.

### Assessment of Blood-Brain Barrier Permeability

BBB permeability was measured by Evans blue (EB) extravasation at day 1 or day 4 after TBI^22,23^. EB (Sigma, St. Louis, MO) with a concentration of 100 mg/kg was injected via the tail vein and allowed to circulate for 1 h. The animals were then perfused with saline via the left ventricle until colorless perfusion fluid appeared from the right atrium. After perfusion and brain removal, the ipsilateral hemispheres were cut into 4-mm-thick sections (2 mm from the frontal pole) before measuring the amount of EB extravasated. The uninjured right hemispheres of sham-operated mice acted as the control. Samples were weighed and then soaked in 50% trichloroacetic acid solution. After homogenization and centrifugation, the extracted dye was diluted with ethanol (1:3), and the amount present measured using a spectrophotometer (Infinite M200, Tecan, Mechelen, Belgium) at 620 nm.

### Histological Evaluation

One, 4, and 28 days following TBI, mice were sacrificed by transcardial perfusion with phosphate-buffered saline (PBS), and then the tissues were fixed with 4% paraformaldehyde. Brains were collected and post-fixed in 4% paraformaldehyde overnight and transferred to PBS containing 30% sucrose for cryoprotection. Coronal sections were cut in a cryostat at 10 μm from the level of the olfactory bulbs to the visual cortex and used for cresyl violet histology, FJB staining, or immunohistochemistry.

#### Cresyl violet staining

The contusion area was quantified using coronal sections stained with cresyl violet at 20 rostral-caudal levels that were spaced 200 μm apart. Sections were digitized and analyzed using a 1.5× objective and Image J software (Image J, National Institutes of Health, Bethesda, MD, USA). The contusion area was calculated using all cresyl violet-stained sections containing contused brain, and the contusion volume was computed by summation of the areas multiplied by the interslice distance (200 μm). The preservation of cerebral tissue was evaluated by the ratio of the volume of the ipsilateral remaining cerebral hemisphere to the volume of the corresponding contralateral cerebral hemisphere.

#### Fluoro-jade B staining

FJB staining was used to label degeneration neurons of the brain. Sections were rehydrated in graded ethanol (50%, 75%, and 100%; 5 min each) and distilled water. Sections were then incubated in a solution of 0.06% potassium permanganate for 15 min, rinsed in distilled water for 2 min, and incubated in a 0.001% solution of FJB (Chemicon, Temecula, CA, USA) for 30 min. FJB staining was quantified on stained sections at the level of 0.74 mm from the bregma. Three sections per animal were viewed and photographed under a microscope. FJB-positive cells were counted by sampling an area of 920 × 860 μm^2^ (FJB staining) immediately adjacent to the cortical contusion margin in 3 randomly selected, non-overlapping fields using a magnification of 20x. The total number of FJB-positive cells was expressed as the mean number per field of view.

#### Immunohistochemistry staining

After quenching of endogenous peroxidase activity and blocking of nonspecific binding with 10% normal goat serum, sections were allowed to react with the primary antibodies (rabbit anti-myeloperoxidase [MPO; a neutrophil marker; 1:1000; Dako 019-19741, Carpinteria, CA, USA] or rabbit anti-Iba1 [a microglia/macrophage marker; 1:1000; Wako 019-19741, Osaka, Japan]) at 4 °C overnight. Further colorimetric detection was processed according to the instructions of a Vectastain Elite ABC Kit (Vector Laboratories, Burlingame, CA, USA) with the use of diaminobenzidine as a peroxidase substrate. The specificity of the staining reaction was assessed in several control procedures, including omission of the primary antibody and substitution of the primary antibody with non-immune rabbit serum. Brain sections from day 1 or day 4 after CCI were used as positive controls for cresyl violet, FJB, MPO and Iba1 staining methods^22,23^.

### Magnetic resonance imaging

Magnetic resonance imaging (MRI) was performed using a 3 TMRI system (TRIO 3T MRI, Siemens MAGNETOM, Germany). Brain edema was assessed by T2-weighted images obtained on day 1 and day 4 post-injury. The parameters for the T2-weighted imaging were as follows: repetition time/echo time = 3500/75 ms, matrix = 125 × 256, field of view = 25 × 43 mm, and section thickness = 1.0 mm. The imaging plane was located across the center of the lesion site. After normalizing image intensities between pre- and post-TBI, areas of hyperintensity represent edema regions. The regions of interest (ROI) were manually outlined by a blinded operator with the ROI tool of the MRI system software (NUMARIS/4, Version syngo MR B17, Siemens MAGNETOM). Edema volumes were assessed from T2-weighted images by summing up the edema area measured from six slices and multiplying by the slice thickness (1.0 mm).

### Western blotting analysis

One and 4 days after TBI, a 4-mm coronal section was taken from the injured area over the parietal cortex and then homogenized by T-Per extraction reagent supplemented with the Halt Protease Inhibitor Cocktail (Pierce Biotechnology, Inc.). Samples containing 30 μg protein were resolved on 12% sodium dodecyl sulfate polyacrylamide gel electrophoresis (SDS-PAGE) and transferred to Immun-Blot® polyvinyldifluoride (PVDF) membranes (Bio-Rad, CA, USA). After blotting, the membranes were blocked for at least 1 h in blocking buffer (Hycell, Taipei, Taiwan), and then the blots were incubated overnight at 4 °C in a solution with antibodies against zonula occludens-1 (ZO-1, 1:200, 61–7300) and claudin-5 (1:1000, 34–1600) procured from Invitrogen (Camarillo, CA, USA). After being washed with PBST buffer, the membrane was incubated with the secondary antibodies for 1 h at room temperature. After being washed with PBST buffer, signals were developed using a Western Lightning ECL reagent Pro (Bio-Rad, California, USA). The gel image was captured using an ImageQuant™ LAS 4000 biomolecular imager (GE Healthcare Life Sciences, Pennsylvania, USA) and analyzed using a gel image system (ImageJ) to estimate the integral optical density of the protein bands.

### Statistics

All data are shown as means ± standard error of the mean (SEM). The Shapiro-Wilk test was first performed to assess the normality of the data. Differences between two groups were performed using Student’s *t* test. A two-way analysis of variance (ANOVA) followed by Tukey’s test was performed to determine the individual and interactive effects of LIPUS on behavioral tasks and the expressions of ZO-1 and claudin-5. The level of statistical significance was set at *p* value ≤ 0.05.

## Results

**Figure 2:**
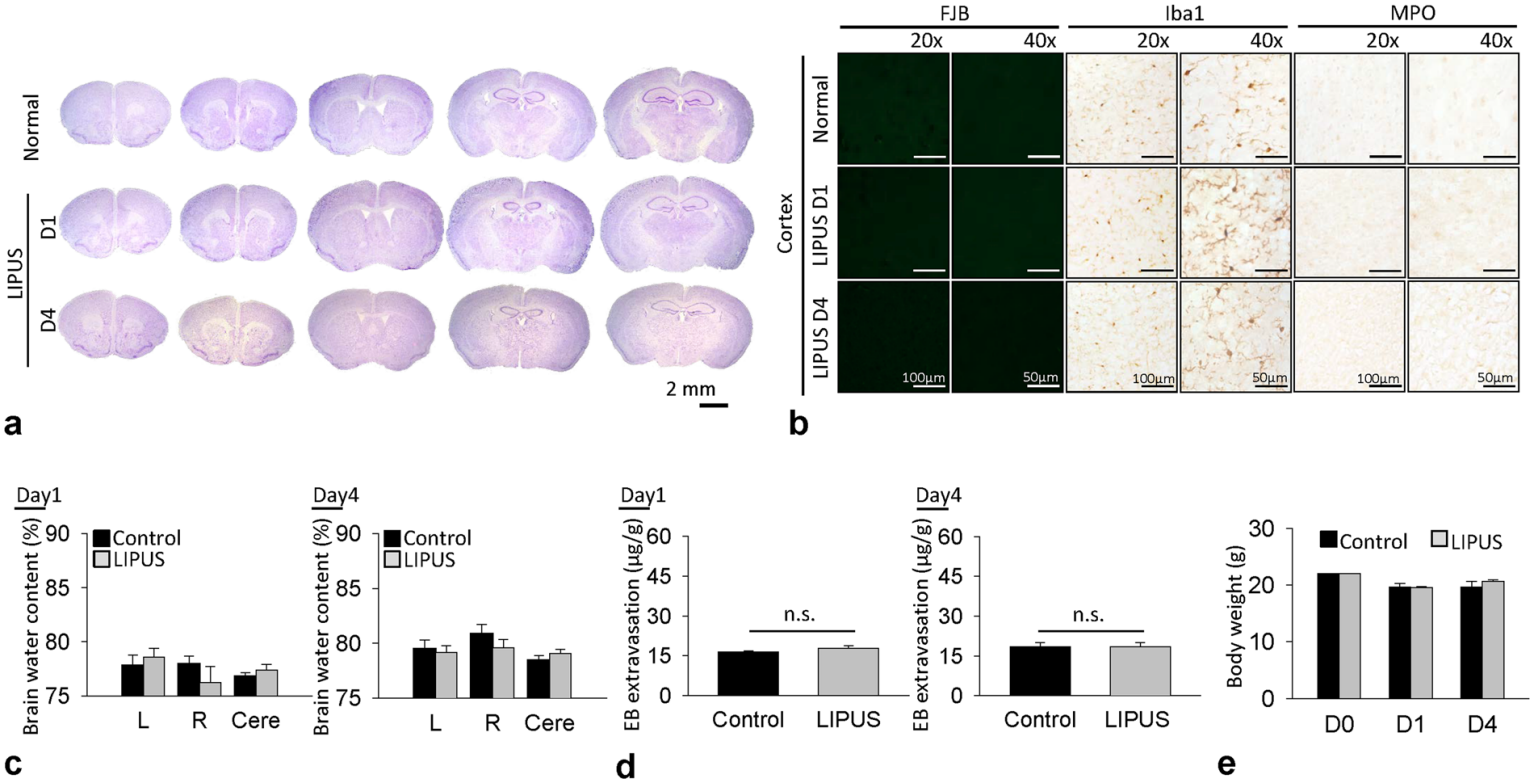
Effect of LIPUS treatment on neuronal damage, inflammatory cells, cerebral water content, and BBB permeability. (**a**) There were no cortical cell losses or intraparenchymal hemorrhages, (**b**) no FJB-positive degenerative neurons, and no activated microglia or infiltrated neutrophils following 1-day or 4-day LIPUS treatment in normal brain. No significant differences were found in (**c**) water content, (**d**) BBB permeability, or (**e**) body weight between sham brain and LIPUS-treated sham brain (*n* = 6).

We first conducted safety experiments to verify whether the LIPUS used in this study would cause neuronal damage or inflammation or affect the cerebral water content or BBB permeability in the normal brain (Fig. 1a). Normal mice served as controls. Animals in the LIPUS group were treated with LIPUS daily for a period of one or 4 days. Cresyl violet staining revealed no cortical cell loss and no intraparenchymal hemorrhages following LIPUS treatment (Fig. 2a). There were also no Fluoro-Jade B (FJB)-positive degenerative neurons detected in the normal brain or the normal brain treated with 1-day or 4-day LIPUS (Fig. 2b). In addition, no activated microglia or infiltrated neutrophils were observed in either of the two groups (Fig. 2b). There was no significant difference in water content between the sham control brain and the sham control brain treated with LIPUS at either day 1 (Fig. 2c) or day 4. Moreover, no significant difference was observed in BBB permeability as assessed by EB extravasation between the sham control group and the LIPUS group at both 1 and 4 days (Fig. 2d). There was also no difference in body weight between the two groups at 1 and 4 days (Fig. 2e). The following experiments were therefore performed in CCI-injured brains with LIPUS stimulation at an acoustic power of 0.51 W.

**Figure 3:**
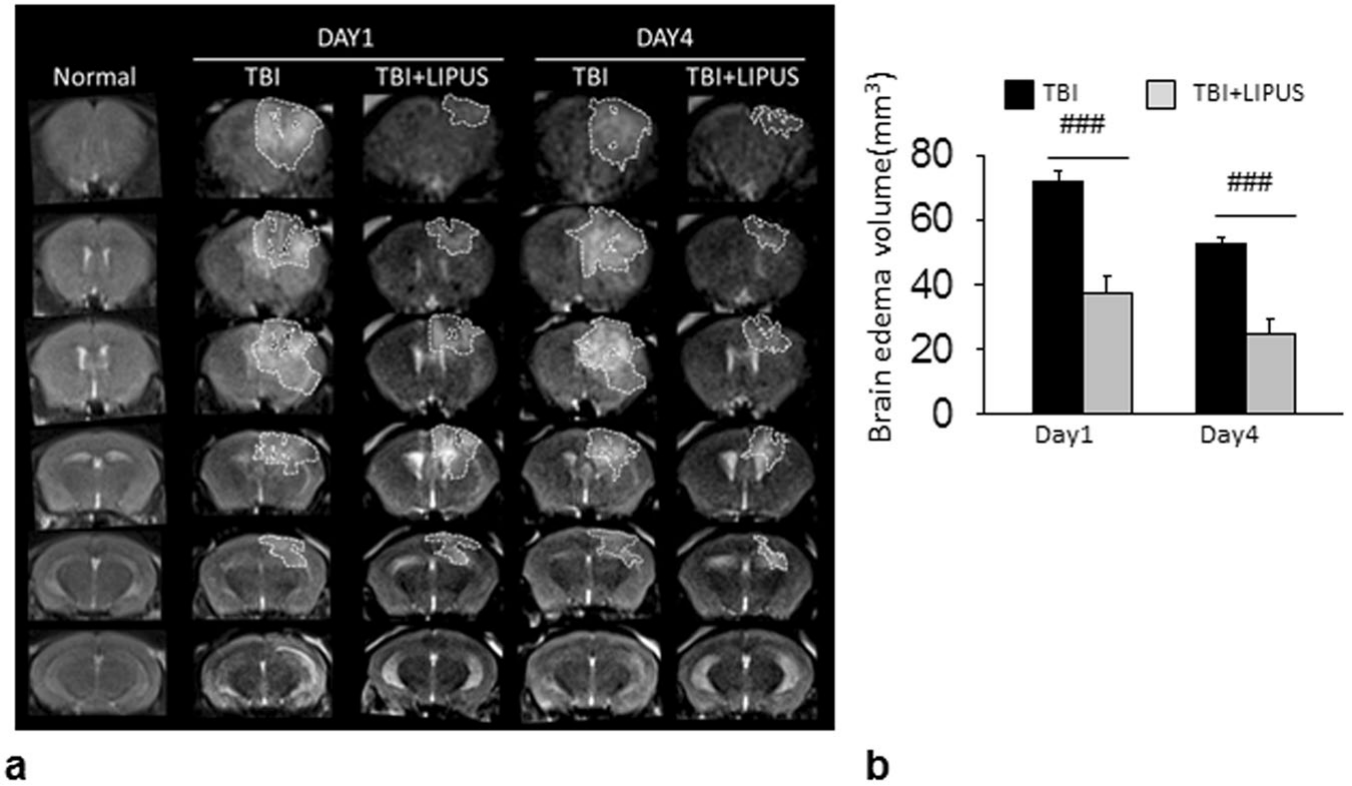
Effects of LIPUS treatment on brain edema in TBI mice. (**a**) Representative T2-weighted MRI images at 1 and 4 days post-TBI. The damaged area is defined as a hyperintense region over the right parietal cortex, indicating edema formation. Dotted line shows location of regions of interest. (**b**) Quantification revealed significantly smaller edema volumes in LIPUS-treated mice compared with non-treated mice at 1 and 4 days. ^#^Denotes significantly different from non-treated TBI group (^###^ *p* < 0.001, *n* = 6).

We then applied MRI to assess the ability of LIPUS to reduce brain damage at 1 and 4 days after CCI (Fig. 3a). Areas of hyper- and hypointensity represent edema and hemorrhage, respectively. Brain edema centered around the contusion site was evident at both 1 and 4 days, and LIPUS significantly reduced T2-weighted lesion volume in injured mice compared with the non-treated group at both day 1 (37.6 ± 4.9 mm^3^ versus 71.8 ± 3.7 mm^3^, *p* < 0.001; Fig. 3b) and day 4 (24.8 ± 4.4 mm^3^ versus 52.8 ± 1.6 mm^3^, *p* < 0.001).

**Figure 4:**
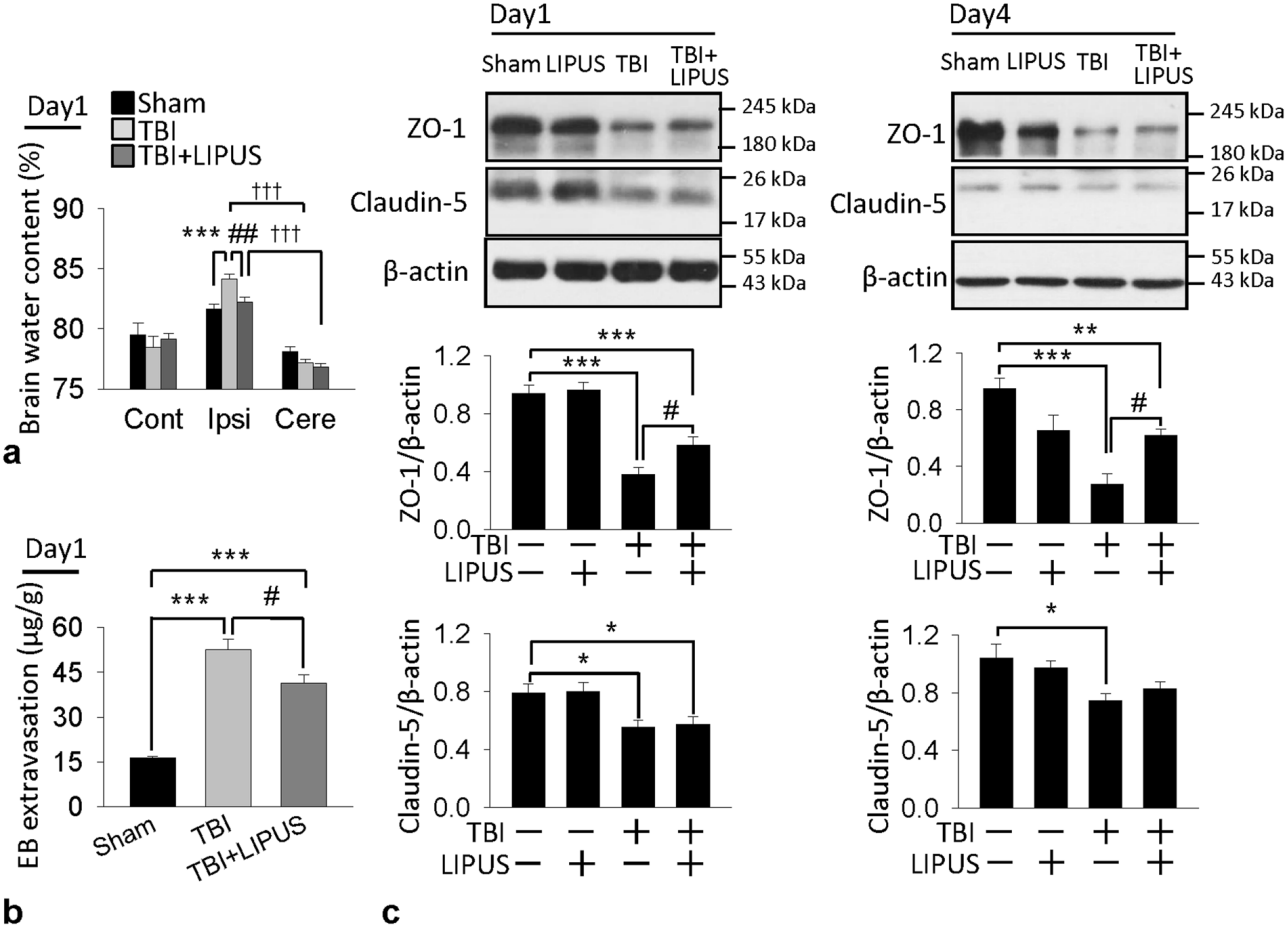
Effects of LIPUS treatment on brain edema, BBB permeability, and tight junction protein expression in TBI mice. (**a**) Brain water content (*n* = 7) and (**b**) leakage of Evans Blue into the brain at 1 day. Ipsi: ipsilateral cortex; Cont: contralateral cortex; Cere: cerebellum (*n* = 5). (**c**) Representative western blots and optical densitometric quantification of ZO-1 and claudin-5 levels at 1 and 4 days post-TBI. LIPUS significantly increased ZO-1 expression at both 1 and 4 days but did not affect claudin-5 expression (*n* = 6). *^,#^, and ^†^denote significantly different from sham, non-treated TBI group, and cerebellum, respectively (*^,#^ *p* < 0.05; **^,##^ *p* < 0.01; ***^,†††^ *p* < 0.001).

These MRI findings were mirrored in the findings regarding brain water content, an indicator of brain edema. Brain water content significantly increased in ipsilateral hemisphere than contralateral hemisphere (all *p* < 0.001) or cerebellum (all *p* < 0.001) in both TBI and LIPUS-treated groups at 1 and 4 days. However, LIPUS caused a significant reduction in the percentage of water content within the ipsilateral hemisphere compared with the TBI group at day 1 (82.5 ± 0.4% versus 84.1 ±  ± 0.3%, *p* = 0.007; Fig. 4a). Because BBB breakdown may result in the accumulation of circulating fluid and lead to brain edema^24^, we further evaluated whether LIPUS treatment could attenuate BBBD at day 1. There was a marked increase in EB extravasation in the ipsilateral hemisphere of the TBI group as compared with the sham group (54.3±3.7 μg/g versus 16.6 ± 0.5 μg/g, *p* < 0.001; Fig. 4b). However, TBI-induced increases in EB content in the ipsilateral hemisphere were significantly attenuated by LIPUS treatment at day 1 post-TBI (54.3 ± 3.7 μg/g versus 41. 3± 3.0 μg/g; *p* = 0.027). We also examined the effects of LIPUS on two major proteins involved in the tight junctions of the BBB, zonula occludens (ZO)-1 and claudin-5. TBI resulted in a significant decrease in both ZO-1 and caludin-5 protein expression at both days 1 and 4 after injury (Fig. 4c). The ZO-1 protein expression was significantly increased following LIPUS treatment. More specifically, the protein expression of ZO-1 in the injured cortex of the LIPUS-treated mice was increased to 152.6% (*p* = 0.036) of that in the TBI group at day 1 and 229.6% (*p* = 0.033) at day 4. However, there was no significant difference in claudin-5 expression between the two groups.

**Figure 5:**
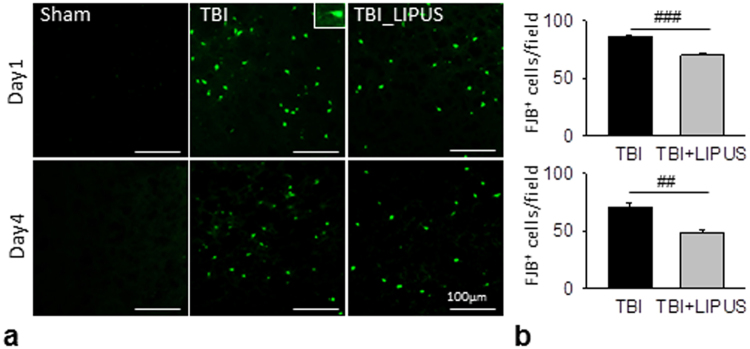
Effects of LIPUS treatment on neuronal degeneration in TBI mice. (**a**) Representative FJB-stained brain sections at 1 and 4 days post-TBI. (**b**) Quantification indicated that LIPUS-treated mice had significantly fewer degenerating neurons than non-treated TBI mice. The total number of FJB-positive is expressed as the mean number per field of view (0.8 mm^2^). ^#^Denotes significantly different from non-treated TBI group (^##^ *p* < 0.01; ^###^ *p* < 0.001, *n* = 8).

We further investigated whether the reduction in brain tissue damage was reflected at the cellular level (Fig. 5a). LIPUS significantly reduced the number of FJB-positive neurons in the contusion margin of the injury core at both day 1 (70.1 ± 2.3 versus 86.7 ± 1.0 cells/field, *p* < 0.001; Fig. 5b) and day 4 (48.3 ± 2.6 versus 70.2 ± 4.4 cells/field, *p* = 0.001).

**Figure 6:**
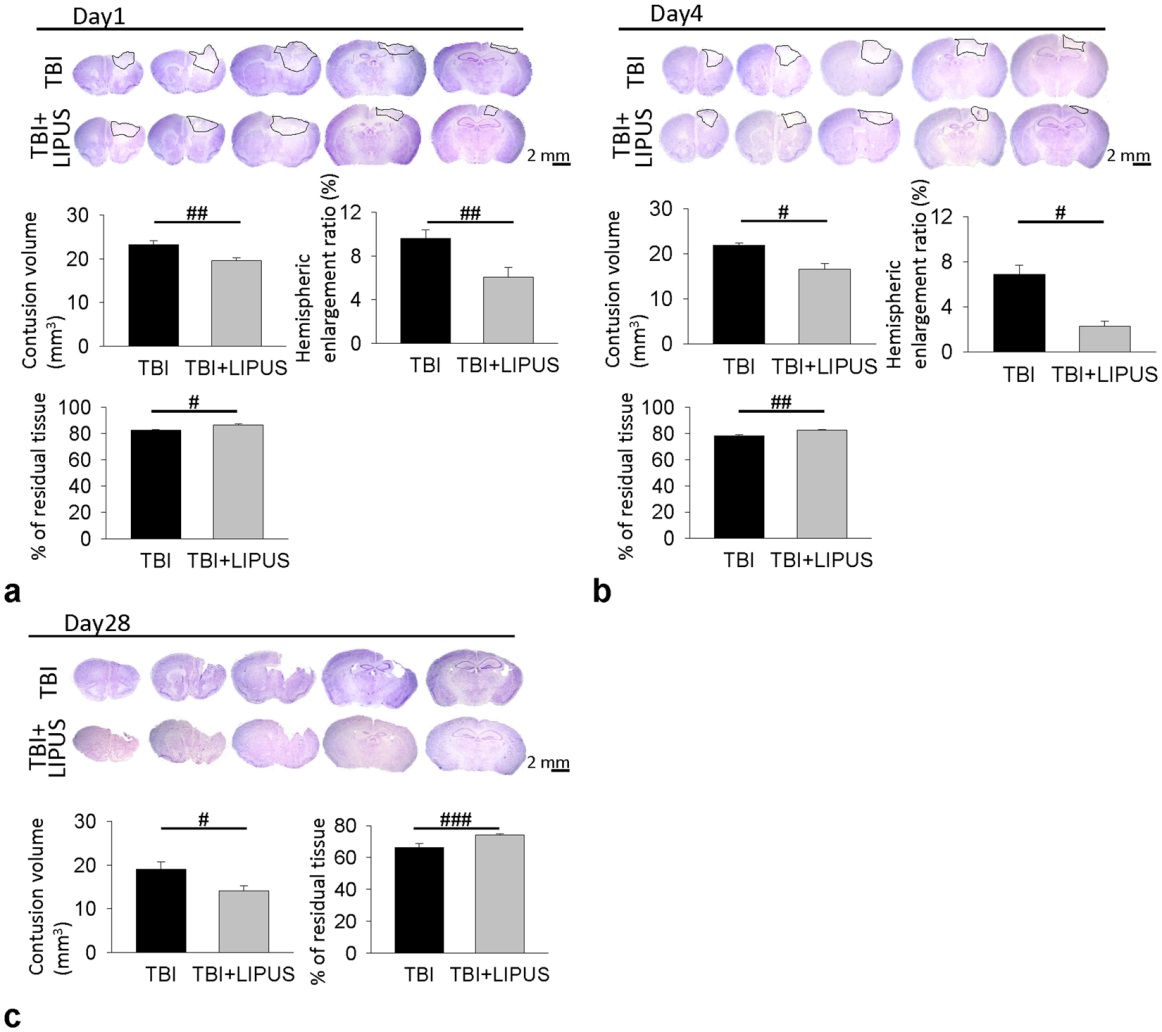
Effects of LIPUS treatment on cortical contusion volume in TBI mice. Representative cresyl violet-stained brain sections at (**a**) 1 (*n* = 8), (**b**) 4 (*n* = 7), and (**c**) 28 (*n* = 6) days post-TBI. Quantification revealed significantly smaller contusion volumes, residual tissue ratio and hemispheric enlargement in LIPUS-treated mice compared with non-treated mice at 1 and 4 days and significantly smaller contusion volumes and higher residual tissue ratio in LIPUS-treated mice compared with non-treated mice at 28 days. ^#^Denotes significantly different from non-treated TBI group (^#^ *p* < 0.05; ^##^ *p* < 0.01; ^###^ *p* < 0.001).

We previously reported that tissue loss during the chronic phase of TBI was associated with neurodegeneration at the acute stage^22,26^, so we analyzed brain tissue damage and neuronal death at 1 and 4 days post-injury. LIPUS significantly attenuated contusion volume to 84.1% of the non-treated TBI-level, that is, from 23.2 ± 0.9 mm^3^ to 19.5 ± 0.7 mm^3^, at day 1 (*p* = 0.005; Fig. 6a) and 74.8% of the non-treated TBI-level, that is, from 21.8 ± 0.8 mm^3^ to 16.3 ± 2.3 mm^3^, at day 4 (*p* = 0.04; Fig. 6b). Likewise, the residual tissue ratios in the LIPUS-treated group were significantly higher than in the non-treated TBI group at both day 1 (86.4 ± 1.2% versus 82.6 ± 0.6%, *p* = 0.012; Fig. 6a) and day 4 (82.5 ± 0.7% versus 78.4 ± 0.9%, *p* = 0.003; Fig. 6b) post-injury. Similarly, hemispheric enlargement was significantly smaller in the LIPUS-treated mice than in the non-treated mice at both day 1(6.0 ± 0.9% versus 9.6 ± 0.8%, *p* = 0.001; Fig. 6a) and day 4 (2.3 ± 0.4% versus 6.9 ± 0.8%, *p* = 0.011; Fig. 6b) post-injury. TBI caused an obvious loss of tissue in the injured hemisphere at day 28 after injury (Fig. 6c). LIPUS significantly reduced contusion volume to 73.8% of the non-treated TBI-level, that is, from 19.06 ± 1.69 mm^3^ to 14.06 ± 1.22 mm^3^, at day 28 (*p* = 0.037). Similarly, LIPUS significantly preserved brain tissue (74.1 ± 0.6% of the contralateral hemisphere) compared with the non-treated TBI group (66.4 ± 2.3%, *p* < 0.001), suggesting neuroprotection of the brain.

**Figure 7:**
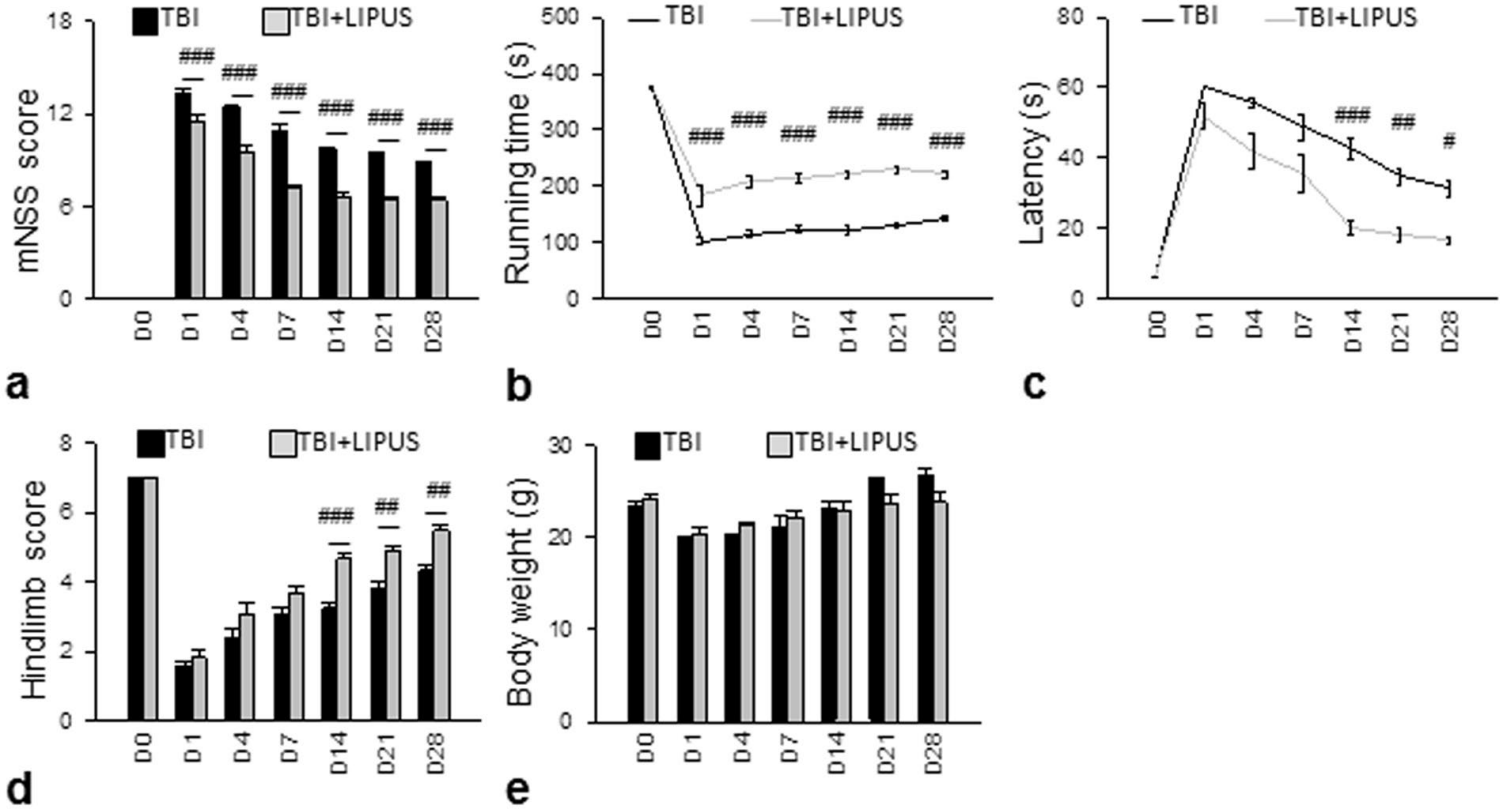
Effects of LIPUS treatment on behavioral outcomes in TBI mice. (**a**) LIPUS significantly reduced mNSS and (**b**) improved the rotarod outcome compared with the corresponding TBI group at test days 1–28. (**c**,**d**) LIPUS significantly improved beam walk performances from days 14–28. (**e**) No significant differences were found in body weight changes. ^#^Denotes significantly different from non-treated TBI group (^#^ *p* < 0.05; ^##^ *p* < 0.01; ^###^ *p* < 0.001, *n* = 12).

To assess whether LIPUS provides protection against TBI, we first performed several sets of behavioral experiments to evaluate the effect of LIPUS on behavioral recovery (Fig. 7). Neurological deficits were itemized and quantified by modified mNSS. The mNSS scores were significantly lower in the LIPUS-treated group than the corresponding non-treated group at test days 1–28 (all *p* < 0.05; Fig. 7a). Mice subjected to TBI presented significant motor dysfunction, as assessed by rotarod and beam walking tests. Compared to the non-treated group, the LIPUS-treated group had better rotarod performance over the whole observation period (all *p* < 0.05; Fig. 7b). Likewise, beam walking latencies were shorter for the LIPUS-treated group from 14 to 28 days (all *p* < 0.05; Fig. 7c). Significant differences in hindlimb score were also observed between the LIPUS-treated and non-treated groups from day 14 to 28 (all *p* < 0.05; Fig. 7d). However, no significant differences were found in body weight change between the two groups (Fig. 7e).

## Discussion

In this study, we provide the first evidence that transcranial LIPUS stimulation improved long-term behavioral outcomes and attenuated brain edema in mice subjected to TBI. BBB disruption and brain tissue damage were also reduced following LIPUS stimulation. Our results suggest that LIPUS stimulation may provide a potential therapy for TBI.

LIPUS has been used clinically in the treatment of bone fractures to accelerate the proliferation and differentiation of osteoblasts^27^. Until recently, there were few reports demonstrating the neuroprotective effects of LIPUS against brain damage in animal models of stroke and neurodegenerative diseases^17,28^. This neuroprotection was associated with an increase of brain-derived neurotrophic factor (BDNF) in the sonicated brain. We found that post-traumatic LIPUS stimulation enhanced functional recovery and reduced cerebral damage in mice, effects which were observed as long as 1 month post-injury. The sustained neuroprotective effect of LIPUS for TBI observed in the present study is important because cerebral injuries arising from ischemic, traumatic, or neurodegenerative insults cause different injury processes and cellular vulnerability patterns^29^. The long-term promotion of functional recovery is also of great clinical relevance, since to date, there are no therapies that can cure the neurological deficits in TBI patients^30^.

Brain edema is one of the major prognostic factors for patients with TBI^6,7^. Previous reports showed that low intensity US reduced brain edema in animal models of water intoxication^31^, weight drop brain injury^18^, and focus ultrasound induced BBBD^20^. In the present study, we showed that LIPUS attenuated brain edema and BBB disruption, as well as an increase in tight junction protein ZO-1 expression. LIPUS maintained ZO-1 expression in the injured brain, suggesting that the protective effect on brain edema may be attributed to the maintenance of tight junction protein. We found that LIPUS increased ZO-1 expression but not claudin-5 loss. Additionally, our previous data showed that the Akt phosphorylation was significantly increased in a time-dependent manner in astrocytes following LIPUS stimulation^32^. It is possible that LIPUS stimulated ZO-1 expression via the activation of Akt since the Akt pathway has previously been shown to be involved in the maintenance of barrier integrity via the regulation of ZO-1 in endothelial cells^33^. A limitation of our study is that the brain region in mice affected by the LIPUS is much larger than in humans. Nevertheless, the basic mechanisms induced by LIPUS may be the same, and the results from this animal study provide key hints for a better understanding and further applications of LIPUS treatment in the patients with TBI. Additionally, the real mechanisms of beneficial effects of LIPUS on TBI are still unknown. Further studies are needed to investigate the detailed molecular mechanisms of neuroprotection caused by LIPUS.

In summary, we demonstrated that post-injury LIPUS treatment significantly reduced contusion volume and improved long-term behavioral outcomes following TBI. The beneficial effects of LIPUS on brain edema are associated with the attenuation of the permeability of BBB and the enhancement of tight junction proteins. Our findings suggest that LIPUS stimulation could be a promising new technique for treating TBI.
